# Commentary: Express saccades and superior colliculus responses are sensitive to short-wavelength cone contrast

**DOI:** 10.3389/fpsyg.2017.00565

**Published:** 2017-04-18

**Authors:** Olivier A. Coubard

**Affiliations:** The Neuropsychological Laboratory, CNS-FedParis, France

**Keywords:** vision, eye movements, saccade, superior colliculus, motor control

## Letter

Recently, Hall and Colby (2016) showed in non-human primates that the superior colliculus (SC) can use short-wavelength-sensitive cone (S-cone) stimuli. The authors used express saccades, a subclass of eye movements which critically depends on the SC (Schiller et al., 1987). Behaviorally they evidenced that S-cone stimuli increase the proportion of express saccades. Physiologically they show that S-cone stimuli yield two neural hallmarks of express saccades in intermediate layer neurons of the SC: larger initial burst of visuomotor activity and stronger preparatory activity, as compared to regular saccades. Taken together with previous reports from other teams leading to the same conclusion (e.g., Chang et al., 2016), these findings have put an end to the dogma according to which the SC does not carry color information. For 14 years, this idea based on the fact that anatomy and physiology had failed to find S-cone input to the SC has echoed two streams of cognitive sciences: naive cognitivism assuming that the brain may work as its inventions, e.g., some thermometer or computer, and Jacksonian neurology suggesting the deeper the brain structure lies the less evolved its function is. In this context the SC has been seen as processing black and white stimuli like the screen of a first generation computer. This view was also incompatible with phylogenetics given that the SC is the most important center for vision in species like inferior vertebrates.

Hall and Colby (2016) grounded their conclusion on the joint analysis of the saccade latency distribution and the neuronal activity in the SC. To study the relationship between behavior and its cerebral condition, the definition of the psychophysical task is critical before entering physiological data. It is the aim of this commentary to discuss this definition. To my view, the demonstration would have gained in strength by excluding from express saccades those short-latency saccades that are not express but early. Which starts with distinguishing between express vs. early saccades. Whilst the former are critically produced by the retino-collicular pathway, early saccades in the continuity of fast regular saccades may still benefit feedforward inputs from other brain areas, including V4, and their possible presence might weaken the conclusion of Hall and Colby (2016). Therefore two properties of saccades have to be examined. First, I would recommend to use in the plot of latency distribution not only a reciprocal scale on the x axis but a probit scale on the y axis to consider only those short latencies lying on a line leftward and parallel to the recinormal distribution of regular saccades, but to exclude short latencies lying on the line whose intercept is 0.5. Indeed early latencies are barely visible on a linear cumulative scale even when a reciprocal time axis is used (Noorani and Carpenter, 2016). Second, the precision of gaze might be weaker in express saccades as compared to early saccades (Hopp and Fuchs, 2004).

Besides technical aspects, express and early saccades may also be different in nature from the theoretical and physiological viewpoints. According to the recently reviewed (Noorani and Carpenter, 2016) Linear Approach To Threshold with Ergotic Rate (LATER) model, express saccades may be due to a LATER unit in which the gain (μ) is larger but the standard error (σ) unchanged as compared to those of regular saccades, leading to a recinormal distribution leftward and parallel to the main one. In contrast, early saccades are thought to be due to a LATER unit whose μ is null and σ higher than that of regular saccades, leading to the recinormal distribution whose intercept is 0.5. In both cases, such LATER units act in parallel to that producing regular saccades. However it is worth noting that the distinction between express and early movements has been made only recently (Noorani and Carpenter, 2011, 2015; Noorani, 2014) and need to be further investigated. Some authors have confused the two responses suggesting evidence for express movements, which were in fact early (e.g., Carpenter, 1994; Merrison and Carpenter, 1995). The latest review (Noorani and Carpenter, 2016) yet struggles to find physiological specificities to express vs. early saccades. Another view is provided elsewhere (Coubard, 2012): if express saccades are due to the failure of the attention-inhibition network running free express movement generators, early saccades may result from specific conditions of attention-inhibition release, where the distance between initial and final thresholds (i.e., θ) and/or the rate of rise (i.e., μ) of LATER units' decisional signal are respectively minimized and maximized. Such contingency can be observed in experts or in participants benefiting from optimal conditions for early triggering.

The distinction between express and early saccades may also help to disentangle the debate on the processes underlying short-latency movement onset—be it preparatory (Hall and Colby, 2016) or not. In neuropsychology, preparatory processes refer to as a voluntary or attention-demanding set of strategic behaviors that sustain the development and maintaining of an optimal processing state prior to the execution of movement (Stuss et al., 1995). In terms of Bayesian models, such definition is synonymous with minimizing θ and/or maximizing μ of the decisional signal. Early movements could be due to preparatory processes and indicate willing behaviors. In contrast, express movements escape volition in a binary, all or nothing way, suggesting that attention-inhibition control has turned off. In this case, decisional mechanisms are bypassed enabling short routes to elicit unwanted behaviors. For saccades, though spatial information may affect express generation, time is more decisive, and spatial effects occur only when favorable timing conditions are met (e.g., the gap effect) so that excitatory modules such as intermediate layer SC neurons increase their activity (Schiller et al., 1987). Here apparent preparation is inherent is released fixation: if one cuts the moorings of a boat (decreased inhibition), it is likely that it moves away (increased activation) though the sailor has not prepared anything. Indeed prefrontal cortex neurons involved in distant inhibition of caudal SC saccade-related neurons exhibit the exact reverse pattern of neural activity after fixation offset with the highest decrease prior to express saccades (Tinsley and Everling, 2002).

## Author contributions

The author confirms being the sole contributor of this work and approved it for publication.

### Conflict of interest statement

The author declares that the research was conducted in the absence of any commercial or financial relationships that could be construed as a potential conflict of interest.
